# *Xylosandrus crassiusculus* (Motschulsky) (Coleoptera: Curculionidae) and Its Fungal Symbiont *Ambrosiella roeperi* Associated with Arecanut Kernel Decay in Karnataka, India

**DOI:** 10.3390/insects13010067

**Published:** 2022-01-06

**Authors:** Shivaji Hausrao Thube, Thava Prakasa Pandian, Anthara Bhavishya, Merin Babu, Arulappan Josephrajkumar, Muddumadiah Chaithra, Vinayaka Hegde, Enrico Ruzzier

**Affiliations:** 1 ICAR—Central Plantation Crops Research Institute, Regional Station, Vittal 574243, Karnataka, India; R.Pandian@icar.gov.in (T.P.P.); Bhavishya@icar.gov.in (A.B.); chaithra.m@icar.gov.in (M.C.); 2 ICAR—Central Plantation Crops Research Institute, Regional Station, Kayamkulam 690533, Kerala, India; merin.babu@icar.gov.in (M.B.); joseph.rajkumar@icar.gov.in (A.J.); 3 ICAR—Central Plantation Crops Research Institute, Kasaragod 671124, Kerala, India; vinayaka.hegde@icar.gov.in; 4 World Biodiversity Association Onlus c/o Museo Civico di Storia Naturale, Lungadige Porta Vittoria 9, 37129 Verona, Italy

**Keywords:** ambrosia beetle, beetle-fungus symbiosis, betel nut, biodiversity, COI, Scolytinae

## Abstract

**Simple Summary:**

The Asian ambrosia beetle *Xylosandrus crassiusculus* is a polyphagous pest that causes extensive damage to several tree crops. The present research reports the incidence of *X. crassiusculus* infestations on areca nuts (betel nuts) in India. Beside the new host plant record, the data provided here represent the first documented case of spermatophagy for this beetle and Xileborini in general. Further, investigations confirmed the association of fungal symbiont *Ambrosiella roeperi* with adult beetles of *X. crassiusculus*. This fungal symbiont was also recovered from the infested galleries present in the arecanut kernel. A preliminary survey showed that the infestation is so far restricted to a limited number of plantations in the Coastal part of Karnataka, India. Incidence of this symbiotic insect-fungus complex in the economic part of arecanut i.e., kernel is of serious concern. In a climate change scenario, this beetle with fungal symbionts may pose a serious threat to arecanut production in India and elsewhere.

**Abstract:**

*Xylosandrus crassiusculus* (Coleoptera: Curculionidae: Scolytinae) is reported causing damage to areca palm plantations (*Areca catechu* L.—Arecaceae) in Karnataka (India). In particular, *X. crassiusculus* has been observed attacking and successfully reproducing on areca nuts; besides the new host plant record, the data provided here represent the first documented case of spermatophagy for this xyleborine beetle. All infestation symptoms of this polyphagous pest were documented and illustrated. The identity of the scolytid, besides morphologically, was confirmed by its DNA barcoding. Eggs, larvae and pupae were found within the galleries of infested kernels. All galleries of the infested kernels were characterized by the presence of whitish to greyish fungal growth. The fungus was identified as *Ambrosiella roeperi*, a known symbiont of *Xylosandrus crassiusculus*. Incidence of this symbiotic insect-fungus complex in the economic part of arecanut, i.e., the kernel, is of serious concern. In a climate change scenario, this beetle with fungal symbionts may pose a serious threat to arecanut production in India and elsewhere.

## 1. Introduction

The granulate ambrosia beetle, *Xylosandrus crassiusculus* (Motschulsky, 1866) (Curculionidae: Scolytinae: Xyleborini) is a polyphagous species native to India and the entire Oriental region [1], that is now widely introduced worldwide [2,3]. It is reported to feed on more than 200 species of trees belonging to 60 families [4]. This species is considered a major pest in fruit orchards, young forest plantations, plant nurseries, and on ornamental trees [5]. In India *Xylosandrus crassiusculus* infests commonly cultivated trees such as *Mangifera indica*, *Eugenia jambolana*, *Dalbergia* sp., *Cinnamoum camphor* and *Shorea* sp. [6]. Most recently, a massive outbreak of *X*. *crassiusculus* on *Syzygium aromaticum* and *Myristica fragrans* was reported from Kerala, India [7]. *Xylosandrus crassiusculus* is also emerging as the key limiting factor for grape production in India [8,9]. This species develops on stressed woody host plants and more frequently on thin-barked deciduous plants [10,11,12,13]. Adult females bore galleries into the plant xylem inoculating mutualistic fungi, which serve as a food source for the developing broods. *Xylosandrus crassiusculus* and the other members of the tribe Xyleborini have been found in close association with species-specific fungal symbionts [14]; these fungi provide the nourishment for larvae and adults in nutrient-poor substrates such as host plant tissues [15]. As of now, various fungal symbionts of *X. crassiusculus* has been identified: *Ambrosiella xylebori* [16,17], *Ambrosiella* sp. 2 [18], *A. xylebori* with unidentified *Fusarium* sp. [19] and *Ambrosiella roeperi* [20], the only species exclusively present in the mycangium. Penetration towards plant tissues and symbiotic fungus settlement results in chewed wood extrusion from entry holes, sap emission, foliage wilting, canopy dieback, and branch and trunk necrosis. The polyphagous nature of this species in association with its dispersal capabilities combined with the great efficiency in locating and colonizing stressed plants make *X. crassiusuculus* management challenging and often ineffective throughout its native and invaded range [21].

Areca palm (*Areca catechu* L.—Arecaceae) is an important cash crop in most Southern Asian countries [22]. The economic part of the palm is known as arecanut or ‘betel nut’ and is mainly used for masticatory purpose in many parts of Asia. India ranks first in area and production of arecanut in the world, with than 16 million people depending on arecanut industry for their livelihood [23]. This nut also plays a significant role as pharmaceutical in Ayurveda (Ancient Indian system of medicine) and in Chinese medicinal practices [24].

During the regular pest and disease surveillance programme, an unusual and widespread infestation on young areca palm plantations was observed in Karnataka, India. The attacks concentrated exclusively on immature areca nuts and showed damaging symptoms consisting in the excavation of tunnels in the nut and emission of frass noodles, similarly to those that ambrosia beetles do on wood. Given the great value that areca nut has for the local economy, great effort was invested to investigate the cause and the incidence of the infestation.

The results of this investigation are presented and discussed below.

## 2. Materials and Methods

### 2.1. Sample Collection, Symptoms Documentation and Pest Distribution

The first immature areca nuts showing damaging or decaying symptoms were collected in two different sites i.e., Nelluru-Kemraje (12°34.487′ N, 75°29.412′ E) and Markanja (12°34.502′ N, 75°29.392′ E), Karnataka (India) during first week of August 2021. All the relevant information viz., age and variety of the infested plantation, cropping pattern and surrounding plantations of the infested sites were collected during the survey. All the visible and concealed damage symptoms were systematically documented by observing infested nuts (*N* = 64 nuts collected from eleven infested palms randomly selected between the two studied plantations). Infested nuts were opened, and the insect specimens were recovered from kernels. Insect galleries presenting fungal mycelia were characterized thoroughly.

Subsequently, the survey was extended to some additional major areca nut growing areas of Karnataka to evaluate the possible extent of the infestation. Observations on percentage of palms infested and percentage of nuts infested were recorded in the surveyed sites. The plantations were also investigated in search of possible *Xylosandrus* attacks on the woody parts of the areca palm. All plantations visited were the standard size of one acre.

### 2.2. Pest Identification

Adult scolytine beetles were morphologically identified using characters and identification keys provided in [25,26]. Four specimens, randomly selected among the previously identified material, were subjected to molecular characterization using the mitochondrial cytochrome c oxidase I (*mtCOI*) gene.

PCR reactions were performed to amplify partial *mtCOI* gene sequences with 25 µL reaction volume containing 2.5 µL of 10X PCR buffer (Thermo Fisher Scientific, Waltham, MA, USA), 1.0 µL of 10 mM dNTPs, 1.0 µL each of forward (LCO1490 5′-GGTCAACAAATCATAAAGATATTGG-3′) and reverse (HCO2198 5′-TAAACTTCAGGGTGACCAAAAAATCA-3′) primers [27] (10 pmol/mL), 1.0 µL of DNA (100 ng/µL), 1.0 µL of Taq DNA polymerase and 18.5 µL of sterile water. Thermocycling (Bio-Rad T100) consisted of an initial denaturation at 94 °C for 3 min, followed by 30 cycles of denaturation at 94 °C for 20 s, annealing at 50 °C for 30 s, extension at 72 °C for 30 s, and final extension at 72 °C for 5 min [22]. The amplified product was evaluated with electrophoresis (Best Lab International Inc., China) using a 1.0% agarose gel [28]. PCR purification (Geneaid, New Taipei City, Taiwan) was done and purified products were sent for Sanger sequencing (Biokart India Pvt. Ltd., Bengaluru, India). Obtained sequences were aligned using BioEdit (Biological sequence alignment editor—Tom Hall. Available online: http://www.mbio.ncsu.edu/BioEdit/bioedit.html, accessed on 20 October 2021) and blasted in the NCBI (National Center for Biotechnology Information. Available online: http://www.ncbi.nlm.nih.gov/, accessed on 20 October 2021) as well as the BOLD database (BOLD Database. Available online: http://www.boldsystems.org/, accessed on 20 October 2021). Sequence similarity was analysed with the available sequences and sequences were deposited in NCBI.

The voucher specimens were deposited in National Insect Museum, ICAR-NBAIR collections under reference number NIM/NBAIR/COL/4921.

### 2.3. Symbiont Fungus Isolation and Characterization

Adult females (*n* = 12) were killed after surface sterilization and the dissected mycangia were inoculated separately on potato dextrose agar (PDA) medium [29]. Simultaneously, insect galleries (*n* = 30) containing fungal propagules were also surface sterilized with 1% sodium hypochlorite solution, inoculated on PDA media and incubated at 28 ± 2 °C for 5 days.

Both symbiont fungi isolated from mycangia and insect galleries were identified using cultural and microscopic descriptions given by Harrington et al. [20]. Molecular characterization of the fungal replicates (*n* = 8) was further confirmed using amplification of ribosomal DNA.

Total fungal DNA was extracted from the mycelial mat using the CTAB method with minor modifications [30]. PCR reactions were performed to amplify the partial rDNA gene sequences with a 25 µL reaction volume containing 2.5 µL of 10X PCR buffer (Thermo Fisher Scientific, Waltham, MA, USA), 1.0 µL of 10 mM dNTPs, 1.0 µL each of ITS1 (5′-TCCGTAGGTGAACCTGCGG-3′) and ITS4 (5′-TCCTCCGCTTATTGATATGC-3′) [31] (10 pmol/µL), 1.0 µL of DNA (100 ng/µL), 1.0 µL of Taq DNA polymerase and 18.5 µL of sterile water. The amplification profile was carried out using a Bio-Rad T100 thermocycler, which was preheated at 95 °C for 3 min, followed by 35 cycles of 95 °C for 30 s, 55 °C for 30 s, 72 °C for 1 min, and final extension at 72 °C for 5 min. The amplified product was visualized using 1% agarose gel, purified using Geneaid PCR Purification Kit (Geneaid, New Taipei, Taiwan) and DNA fragment was sequenced by Sanger’s sequencing (Biokart India Pvt. Ltd., Bengaluru, India).

The obtained sequences were aligned using BioEdit (Biological sequence alignment editor—Tom Hall. Available online: http://www.mbio.ncsu.edu/BioEdit/bioedit.html, accessed on 20 October 2021) and compared with the available sequences in NCBI (National Center for Biotechnology Information. Available online: http://www.ncbi.nlm.nih.gov/, accessed on 20 October 2021). The obtained sequences were deposited in the GenBank.

## 3. Results

### 3.1. Ambrosia Beetle Identification

**Figure 1:**
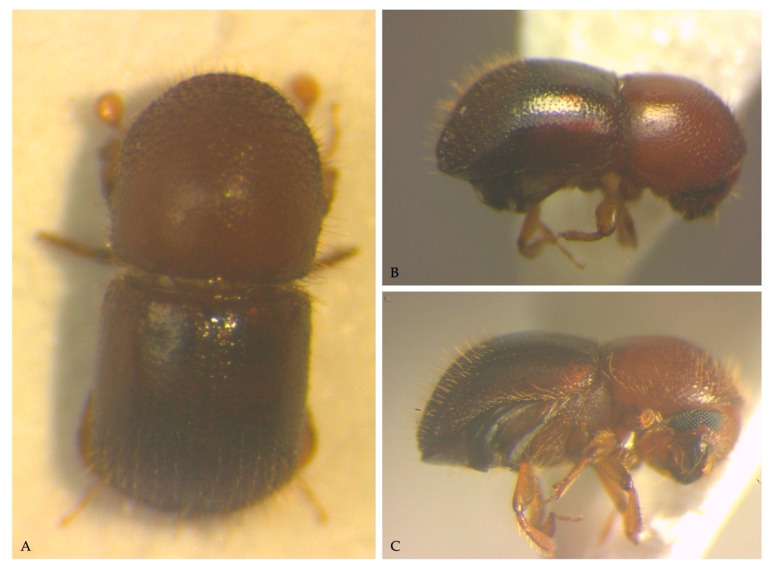
*Xylosandrus crassiusculus*, specimens collected from galleries on areca nuts: (**A**) dorsal view, alive specimen; (**B**) lateral view; (**C**) ventro-lateral view.

All the scolytid beetles collected inside areca nuts were identified as *Xylosandrus crassiusculus* (Scolytinae: Xyleborini) (Figure 1C). The specimens ranged between 2.5–2.8 mm in size, and presented all the typical features of the species: color ferruginous with elitral declivity darker and matte, posterolateral margins of elytra carinate to interstriae seven; pronotum rounded in dorsal view, as long as wide, smooth on the basal half; mycangial tuft located on the pronotal base [24].

Molecular identification of the beetle was confirmed by amplifying 771 bp of the mtCOI gene. The nucleotide sequence of the mtCOI gene was deposited in GenBank (Accession numbers MZ895370-MZ895373) and showed 99.29% similarity with *X. crassiusculus* (GenBank accession No. KX035196) submitted by Natural History Museum, London, United Kingdom.

### 3.2. Symbiont Fungus Identification

**Figure 2:**
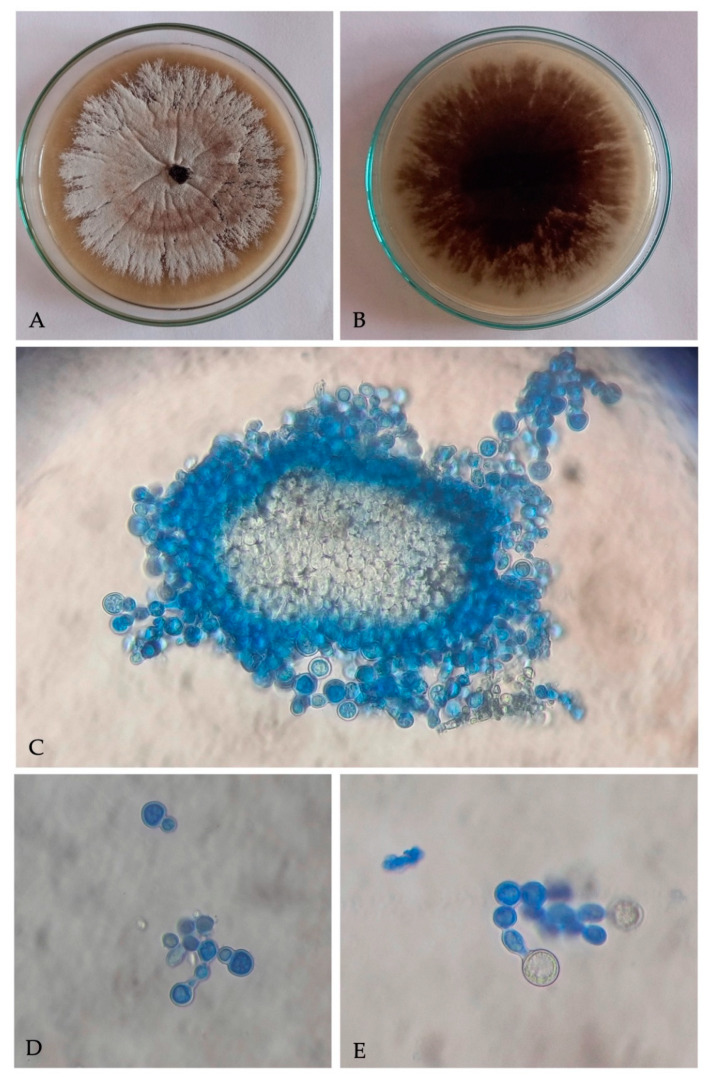
Culture of *Ambrosiella roeperi*: (**A**) upper surface of culture plate; (**B**) under surface of culture plate; (**C**) sporodochial mass isolated from mycangia; (**D**) branched conidiophores with terminal aleurioconidia; (**E**) developing aleurioconidia on swollen conidiophore cells.

A filamentous fungus with cultural and microscopic characters in conformity with the descriptions of *Ambrosiella roeperi* [20] was consistently isolated from *X. crassiusculus* females and insect galleries. Fungal colonies on PDA, appear smoke gray at periphery, whitish to vinaceous buff at center, and 6–8 d culture emits strong sweet odor (Figure 2A). Underside of colony appears dark brown to black (Figure 2B). Microscopic characteristics of *A. roeperi* isolated from female mycangia and galleries observed as the presence of sporodochial mass; branched conidiophores with terminal aleurioconidia and developing aleurioconidia on swollen conidiophore cells (Figure 2C–E).

No other fungus was isolated from mycangia of adult females. However, all the inoculated galleries yielded a filamentous fungus matching the descriptions of *A. roeperi*, except for one gallery where *Fusarium* sp. was isolated. Isolation of a single *Fusarium* colony during the study could be a secondary contamination and no further efforts were taken to characterize this fungus.

Molecular identification of the fungal replicates (*n* = 8) was confirmed by amplifying 580 bp of the ITS gene. The nucleotide sequences of the ITS gene were submitted to GenBank (accession number MZ854170-MZ854177) and showed 100% similarity with *A. roeperi* (GenBank accession No. MK118927) submitted from the IFAS, University of Florida, Gainesville, FL, USA.

### 3.3. Infestation Symptoms, Damage and Incidence

**Figure 3:**
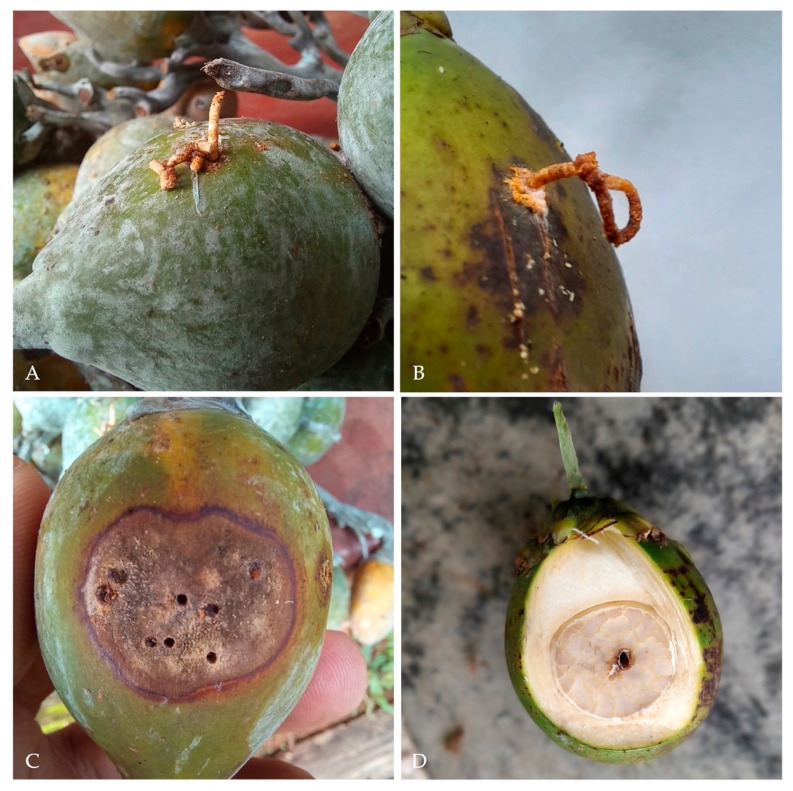
Areca nuts showing symptoms of the attack by *Xylosandrus crassiusculus*. (**A**,**B**) nut showing the typical frass noodle produced by the boring activity of the beetle; (**C**) nut presenting multiple entrance holes in association to a dark staining of the exocarp; (**D**) longitudinal section illustrating one *Xylosandrus crassiusculus* piercing through the nut kernel.

**Figure 4:**
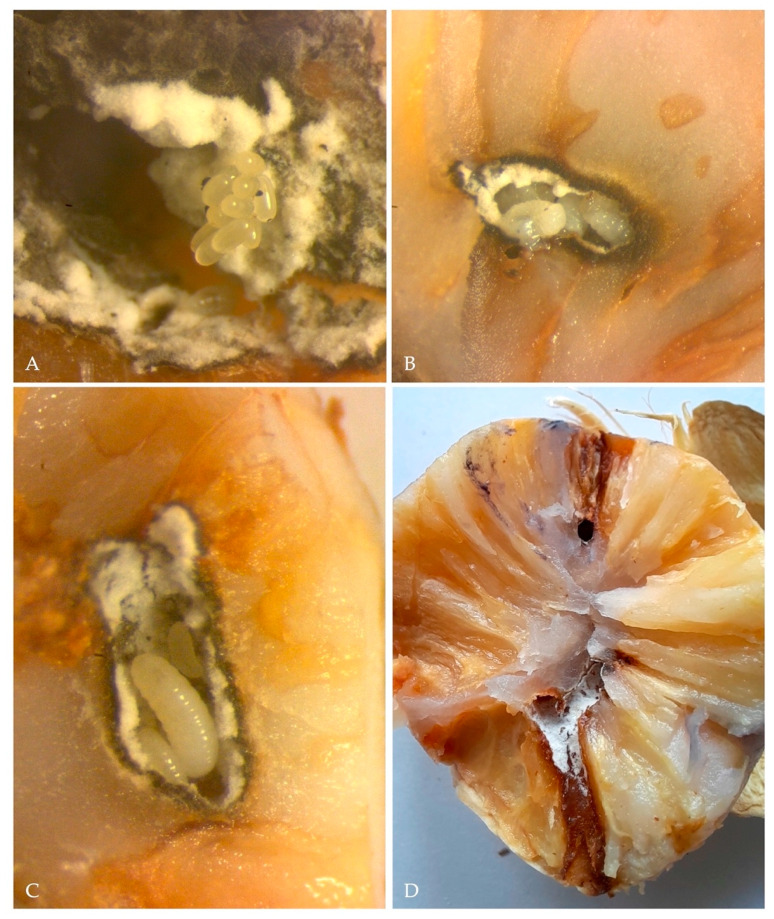
(**A**) eggs of *X. crassiusculus*; (**B**) larvae of *X. crassiusculus* found in one of the galleries observed; (**C**) *X. crassiusculus* larvae at different development stages in association with *A. roeperi* hyphae; (**D**) longitudinal section illustrating *X. crassiusculus* galleries and kernel staining due to the onset of *A. roeperi*.

In the majority of the infested nuts (*n* = 60) only a single adult per fruit was observed while four nuts possessed two to three adults simultaneously. Each female bored a single gallery starting from the exocarp and penetrated through the mesocarp up to the nut kernel. This boring process resulted in the extrusion of the frass in the typical noodle-like shape, almost identical to that this species usually caused on woody trunks (Figure 3A,B). Gallery length varied from 5.0 to 16.0 mm, with an entry hole ranging from 1.06–2.39 mm (Mean ± SE = 1.46 ± 0.11 mm, *n* = 30) (Figure 3C,D). The gallery produced presented a series of chambers most of which hosted both eggs, larvae and pupae; furthremore, all galleries presented a black staining of the nut tisues and the profuse growth of a greyish fungal colony (*Ambrosiella roeperi*) (Figure 4A–C). The onset of *Ambrosiella roeperi* initiated a progressive decay of areca nut kernels, which caused the degeneration of the nut, compromising its maturation and consequently its marketability (Figure 4D).

**Figure 5:**
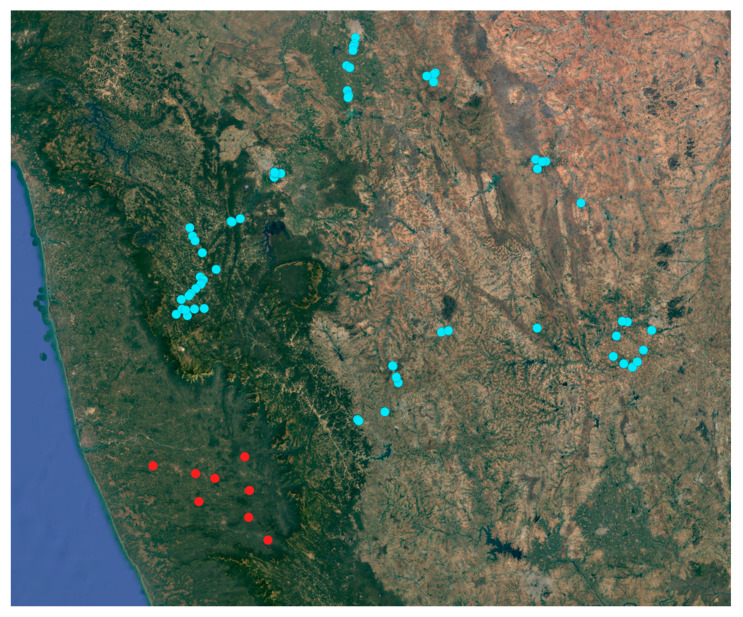
Map of the areca palm plantation surveyed; sites where no infestation has been detected (blue circle), sites where damages caused by *X. crassiusculus* have been observed.

**Figure 6:**
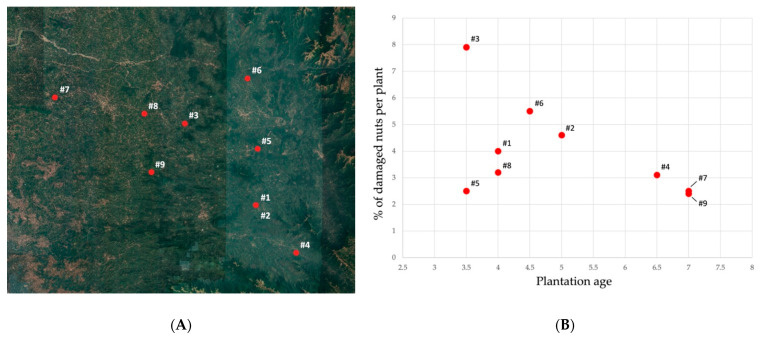
(**A**) Map of the plantations presenting damages caused by *X. crassiusculus*, numbered following the order given in Table 1; (**B**) Plot illustrating the percentage of the damaged nuts on infested plants in association with the age of the areca palm plantation.

After the first detection of *Xylosandrus crassiusculus* in the first two localities (mentioned above), 30 additional plantations in Coastal Karnataka were surveyed (Figure 5); as a result 9 plantations out of 30 presented visible infestation symptoms (Figure 6, Table 1). These infested plantations had a plant density of 550 plants per acre and the number of infested plants was 9.3 per acre on average; in each infested palm the 4% of the total nuts presented attacks by *X. crassiusculus*. In terms of total areca nut production per acre, the attacks represented 0.07% on average. It was interesting to note that in the sampled plantations, *Xylosandrus crassiusculus* attacks only occurred on young plantations with an age between three and seven years (Figure 6). No attacks of *Xylosandrus crassiusculus* were recorded on woody parts of the areca palm, in all the sites surveyed.

## 4. Discussion

Ambrosia beetles are widespread colonizers of physiologically stressed or weakened plants [32] and *X. crassiusculus* preferentially colonizes apparently healthy or recently stressed trees [33]. In our study, we noticed that this species not only affected juvenile and apparently healthy areca palms, but that the attack was limited, contrary to expectations, only to the immature nuts while no other woody parts of the plant were infested.

This observation was indeed interesting because (1) *Areca catechu* was a new host plant and (2) for the first time spermatophagous behavior was observed in *X. crassiusculus*, and possibly among all Xyleborini. It was difficult to explain how this species has come to adopt a spermatophagous behavior, but the fact that the attacks were observed in multiple non-adjacent areas, and that living and prolific infestations have been found within areca nuts, would seemed to support the fact that this was not a unique event but a normal reproductive strategy.

*Areca catechu* is native to the Philippines and widely cultivated in the Oriental region, the geographic area in which *X. crassiusculus* is also native and widely distributed. Given the economic importance of areca nuts to the oriental market, it appeared very unusual that no damage caused by *X. crassiusculus* had been documented so far. Since the attacks were recorded only in Karnataka, where the areca palm was introduced, it is plausible that spermatophagy may be a biological feature belonging to that specific, local population of *X. crassiusculus* and possibly associated with a particular strain of *A. roeperi.*

*Xylosandrus crassiusculus* is a species known to respond to the ethanol emitted by stressed plants [12,34] and the fact that it attacked immature fruits might be due to ethanol content or emission by arecanut during its ripening process.

It is possible that in agroecosystems devoted to monoculture (such as the ones investigated), the historical lack of hostplants may have selected plastic scolytid-symbiont relations, capable of exploiting unusual substrates/host; alternatively, it is plausible that these monocultures generate environmental conditions that promote survival strategies that are hardly favorable in other situations. Scolytids have already been shown to start settlements on substrates of non-botanical origins simply by responding to one or more olfactory stimuli [35,36] indicating that the limiting factor in the success of a species is to identify the best substrate on which to grow the symbiotic fungus. In our specific context, the areca nut may have become the best host for *A. roeperi* because its general structure and constitution resembles the woody tissues where generally *Ambrosiella* fungi develop. In fact, the areca nut husk is predominantly composed of cellulose and varying proportions of hemicelluloses and lignin. The total hemicellulose content varies with the development and maturity, decreasing during the ripening process; contemporarily the lignin content proportionately increases with the development until maturity [37].

The causes that determined the onset of this infestation remain unknown and it will be necessary to investigate further in this regard, especially if the incidence of damage and the extent of the affected areas increases in the near future. The data collected here in preliminary form seem to indicate a relatively low damage density in this phase, with only young plantations infested. However, since areca palms are already subject to innumerable pests and diseases that compromise their productivity and survival [38,39,40,41], a further loss of product, albeit modest, could have important effects on the farming communities that depend from it. To date, very little is known about possible effects of climate change on the biology and behavior of *X. crassiusculus*; however, the important changes taking place could have relevant repercussions on the impact of this species both on a small and large scale [42,43].

Moreover, occurrence of *Ambrosiella* inside the kernels is likely to hamper the market quality and economic return of the infested product. Furthermore, Indian growers store their dry kernels for long periods (up to three years) in warehouses awaiting good market prices [44]. Under such situations, undetected infested nuts could act as a source for future infestations, facilitating the spread of this pest and the fungus in new areas.

The fact that all the nuts presenting the attacks were immature would lead to exclude that the infestation could develop on ripe areca nuts during storage. However, this possibility needs to be carefully evaluated.

## 5. Conclusions

The findings here presented further demonstrate the great ecological plasticity of *X. crassiusculus* and its symbiont *A. roeperi* in successfully adapting to sub-optimal conditions for their proliferation. The capability of *X. crassiusculus* to infest parts of the plant other than trunk and branches, particularly affecting young plants in good health, represent a potential emerging threat to arecanut production in Karnataka, especially in those areas where plantation renewal is underway. The fact that *X. crassiusculus* can behave as a spermatophagus species further increases the difficulty in its already complex pest management. Given the novelty and uniqueness of the find, it will be necessary to investigate the environmental and anthropogenic factors that determined the onset of the infestation and define a monitoring and decision plan that can help limit the damage through the implementation of an IPM strategy that takes into account the needs of producers, but that is at the same time sustainable for the environment.

## Figures and Tables

**Table 1:** Localities where *X. crassiusculus* attacks were recorded during the survey in association with damage incidence.

Location	Palms Age (Years)	*n*° Infested Palms	*n*° Damaged Nuts/Total Nuts on Infested Palms	% of Damaged Nuts on Infested Palms	% of Damaged Nuts
12°34.487′ 75°29.412′	4	7	11/272	4	0.05
12°34.502′ 75°29.392′	5	12	18/384	4.6	0.08
12°43.563′ 75°21.334′	3.5	27	68/856	7.9	0.31
12°29.201′ 75°33.987′	6.5	2	3/95	3.1	0.01
12°40.758′ 75°29.604′	3.5	11	8/320	2.5	0.04
12°48.570′ 75°28.458′	4.5	2	6/109	5.5	0.04
12°46.472′ 75°06.575′	7	12	14/555	2.5	0.06
12°44.664′ 75°16.736′	4	5	6/185	3.2	0.03
12°38.160′ 75°17.535′	7	6	6/244	2.4	0.03

## Data Availability

The data presented in this study are available upon request from the corresponding author.
